# Introducing Maternal Oral Health As Global Health And Public Health Agenda

**DOI:** 10.1093/eurpub/ckac129.257

**Published:** 2022-10-25

**Authors:** H Lee

**Affiliations:** Oral Health Workgroup, WFPHA, Geneva, Switzerland

## Abstract

**Background:**

Achieving and maintaining good oral health during pregnancy is essential for both the oral and overall health of expecting mothers and their young children. Dental caries can negatively affect daily activities including eating, speaking, and social interaction, and when expecting mothers have active dental caries, the risk for dental caries in these children becomes higher. Another common oral disease, periodontitis, was found to be associated with systemic conditions, including cardiovascular disease, respiratory disease, diabetes, and potentially adverse birth outcomes.

**Importance:**

Some studies also showed the systemic impact of periodontal disease and the related pathogens that can lead to systemic inflammation and adverse birth outcomes. A mother's oral health status, her oral health knowledge, and beliefs also have been shown to significantly affect diet and home oral hygiene practice among her children.

**Problem:**

Despite the importance of mother's oral health and oral health knowledge to prevent dental caries in young children, maternal oral health is much neglected in primary and prenatal health discussion. This is even more significant in developing countries where oral health care infrastructure and workforce are inadequate. With increase in sugar consumption growing worldwide with this lack of oral health care infrastructure, oral diseases prevention and oral health promotion are the key to achieve oral health parity among the maternal and child population.

**Solution:**

In this presentation, the author will discuss literature on maternal oral health and how to position oral health as an essential part of perinatal care along with WHO oral health initiative set for 2030. This is an introduction presentation for this workshop to set a stage and make a case why maternal oral health is a critical topic to discuss in global health and public health perspectives.

